# Characteristics of soil organic nitrogen fractions under vegetation restoration in karst areas

**DOI:** 10.1038/s41598-025-08232-7

**Published:** 2025-08-01

**Authors:** Yingge Shu, Xianghuan Gong, Jianghong Wu

**Affiliations:** College of Agriculture, Guizhou University, Guiyang, 550025 China

**Keywords:** Vegetation restoration, Organic fractions of nitrogen, Environmental factors, Restoration ecology, Forestry

## Abstract

To understand the characteristics of soil organic nitrogen fractions under vegetation restoration in karst areas, soil samples from grassland (5-year-old), shrub-grassland (15-year-old), shrubland (20-year-old) and forestland (40-year-old) were taken as the research objects. The Bremner acid—hydrolysis method was used to determine the soil nitrogen fractions, and the correlations between them and soil physical and chemical properties were analyzed. The results showed that with the increase of soil depth, the mass fractions of soil total nitrogen (TN), non-acid-hydrolyzable nitrogen (AIN), acid-hydrolyzable nitrogen (TAN) and their various components all showed a downward trend. The mass fractions of various organic nitrogen components and their proportions in TN from large to small were as follows: AIN, acid-hydrolyzable unknown nitrogen (TUN), acid-hydrolyzable ammonia nitrogen (AMN), acid-hydrolyzable amino acid nitrogen (AAN), acid-hydrolyzable amino sugar nitrogen (ASN). TUN and AMN were likely the main sources of available nitrogen for plant uptake and utilization. The contents of both TAN and AIN increased significantly with the increase of TN content (*P* < 0.001). However, vegetation restoration increased the proportion of AIN and decreased the proportion of TAN. Among them, the 15-year-old shrubland had outstanding soil nitrogen-supplying capacity, while there was no significant difference in soil nitrogen-supplying potential among different restoration years. Soil organic carbon, total nitrogen and ammonium nitrogen were all extremely significantly (*P* < 0.001) positively correlated with soil organic nitrogen fractions. The changes in the characteristics of organic nitrogen fractions affected the soil nitrogen supply and storage capacity. The results of this study can provide a reference for nutrient management during the restoration of degraded soils.

## Introduction

Soil organic nitrogen (SON) accounts for more than 90% of the total soil nitrogen (TN). It acts as both a source and a reservoir for soil mineral nitrogen^1^. The content of easily mineralizable organic nitrogen fractions determines the nitrogen supply capacity of the soil, which in turn significantly affects the growth of above-ground vegetation and the functioning of the ecosystem^2,3^. Based on the chemical forms of organic nitrogen, soil organic nitrogen can be divided into non-acid-hydrolyzed nitrogen (AIN) and acid-hydrolyzed total nitrogen (TAN). The acid-hydrolyzable nitrogen consists of acid-hydrolyzable amino acid nitrogen (AAN), acid-ammoniacal nitrogen (AMN), aminoglycan nitrogen (ASN), and acid-unknown nitrogen (TUN)^4^. Among them, AAN is an important intermediate product in the mineralization of organic nitrogen into inorganic nitrogen. It is closely related to microbial metabolic activities and can be used to characterize the nitrogen supply potential of soil^5^. AMN is mainly in the form of fixed ammonium and is the main direct source of mineralizable nitrogen in soil. The level of its content directly affects the nitrogen supply potential of soil. ASN is a relatively stable type of organic nitrogen, originating from the cell—wall components of microorganisms, which reflects the process of nitrogen assimilation, absorption, and utilization by soil microorganisms^6,7^. TUN refers to the nitrogen-containing compounds that remain unidentified during the acid—hydrolysis process. AIN is a complex compound formed by the condensation of amino acids and amino sugars, with stable properties^8^. Therefore, exploring the content and accumulation characteristics of soil organic nitrogen fractions is of great significance for revealing the availability of soil nitrogen and tapping into the nitrogen supply potential.

This research is crucial for enhancing soil fertility, understanding nitrogen cycling, and protecting the environment, and has long attracted high attention from researchers. Existing studies have mostly focused on the impacts of factors such as fertilization and irrigation^9,10^, vegetation restoration patterns^11–13^, and different land—use types^14–16^ on soil organic nitrogen fractions. Some studies have found that vegetation restoration may either reduce soil organic nitrogen fractions or have no significant impact^17^. Evidently, there is still considerable uncertainty regarding the changes in soil organic nitrogen fractions during the vegetation restoration process. This may be the result of the combined effects of factors such as regional type, restoration duration, vegetation restoration type, soil depth, and soil physical and chemical properties^18^. Therefore, a profound understanding of the evolution characteristics of soil organic nitrogen fractions during the dynamic process of vegetation restoration can provide theoretical support for formulating reasonable soil nitrogen management strategies for ecosystems.

The karst region features a fragile and sensitive ecosystem^19^. Human activities have triggered vegetation degradation and soil erosion, exerting severe negative impacts on the regional ecosystem^20^. Currently, the conversion of farmland to forest has been implemented to combat rocky desertification^21^. To effectively control rocky desertification in the karst region, it is crucial to gain an in—depth understanding of the dynamics of soil organic nitrogen (ON), which can provide strong support for optimizing soil management and promoting crop growth. In this study, the space-for-time substitution method was adopted^22^ to explore the distribution of soil organic nitrogen fractions in different soil layers and the regulatory effects of environmental factors. Taking cultivated land as a reference, grasslands, shrub-grasslands, shrublands, and forestlands with restoration durations of 5 years, 15 years, 20 years, and 40 years were selected, and soil samples from the 0–40 cm soil layer were collected^23^. The aim of this study was to quantify the differences in soil organic nitrogen fractions under different vegetation restoration durations, and determine the regulatory effects of environmental factors. The results of this study can provide scientific references for optimizing land—use structure and land management.

## Materials and methods

### Study area

**Fig. 1:**
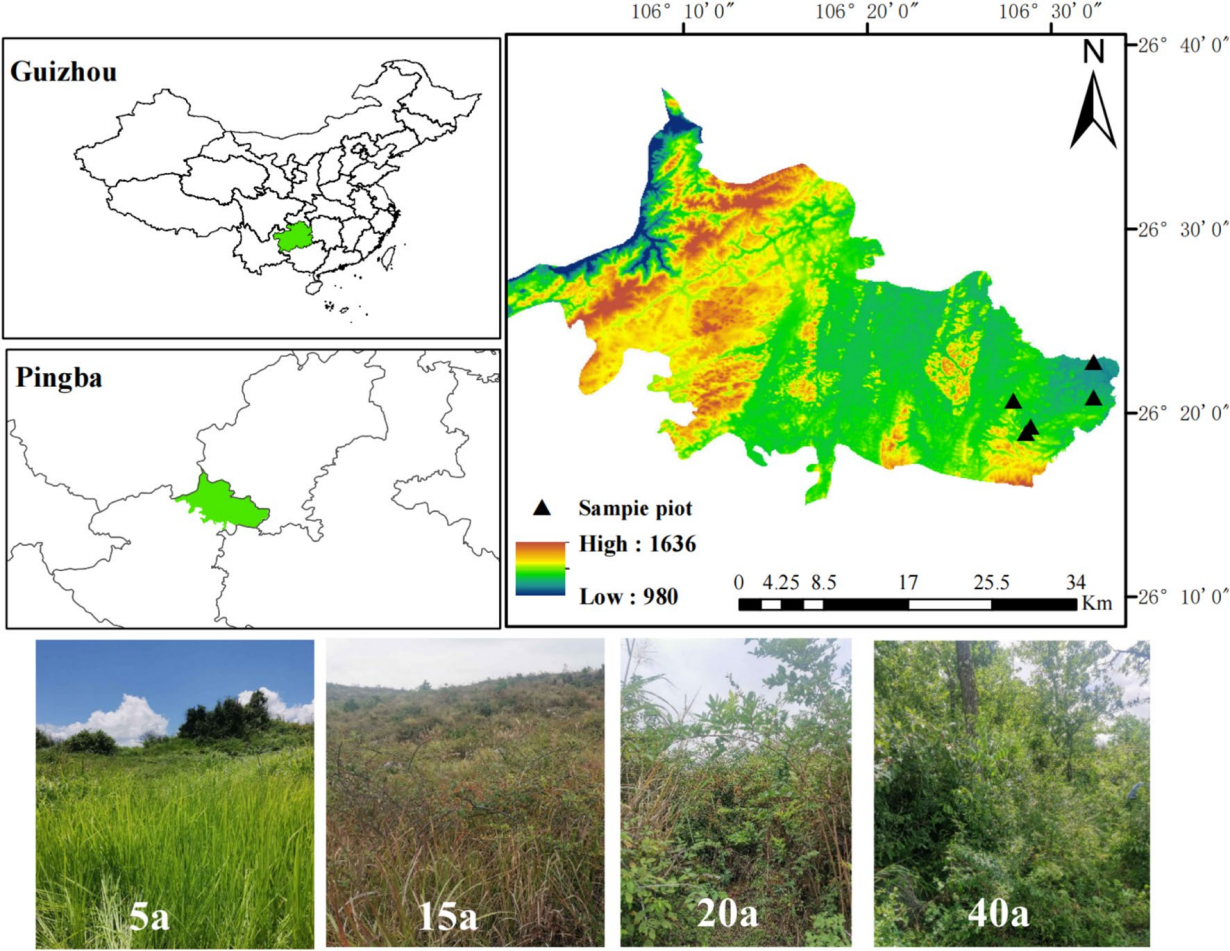
Details of sample site distribution. This map was created by the authors using ArcGIS software (version 10.8) (https://www.arcgis.com/).

Pingba District (26°15′–26°37′40″N, 105°59′20″–106°33′43″E), Anshun City, Guizhou Province, falls in a subtropical humid monsoon climate zone and has an elevation and average annual temperature of 963–1645.6 m and 13.3 °C, respectively. The study area has complex topographic conditions, characterized by typical karst landscapes and diverse types of restored vegetation. Parent rock of the study area is dominated by limestone, whereas the soil is mainly calcareous. Maturity of restored vegetation was determined through a combined approach of examining Google Earth images, fieldwork, and visits to local villages (Fig. 1).

### Selection of sample plots and sampling

The present study identified different types of restored vegetation sample plots with largely common environmental conditions, focusing on vegetation restoration type, topography, and soil type. These selected restored vegetation types were grassland, shrub-grassland, shrubland, and forestland with restoration maturities of 5 years, 15 years, 20 years, and 40 years, respectively, whereas adjacent cultivated land was used as a control (CK). The dominant vegetation type in grassland was Leucaena [*Imperata cylindrica (L.) Beauv*]; that in shrubland included pyracantha (*Pyracantha fortuneana*), artemisia (*Artemisia annua*), wild berry (*Rubus *et al*.*), and wild peppercorn (*Zanthoxylum simulans*); that in forestland included Park and Rowan (*Catalpabungei C. A. Mey, Celtis sinensis Pers*); that in arable land was maize (*Zea mays*) (Table 1). Using the S-shaped sampling method, three soil profiles were excavated repeatedly in each sample plot, resulting in a total of 15 profile excavations. Within the depth range of 0–40 cm, the soil was divided into five layers, namely 0–5 cm, 5–10 cm, 10–20 cm, 20–30 cm, and 30–40 cm. Subsequently, a total of 75 soil samples were collected layer by layer. After collection of soil samples, impurities visible to the naked eye, such as roots, gravel, plants, and animal debris were removed, and the samples were filtered through a sieve.

**Table 1:** Summary description of the sample site.

Sample type	Recovery years/a	Altitude/m	Longitude and latitude	Major vegetation
Farmland	0	1211	26°20′52″N, 106°32′18″E	*Zea mays*
Grassland	5	1217	26°20′59″N, 106°32′18″E	Imperata cylindrical
Shrub-grassland	15	1285	26°20′5″N, 106°27′56″E	Imperata cylindrical
Shrubland	20	1289	26°18′54″N, 106°28′39″E	*Pyracantha fortuneana*, *Artemisia annua*, *Rubus idaeus* L., *Zanthoxylum simulans*
Forestland	40	1223	26°19′19″N, 106°29′6″E	Catalpabungei C. A. Mey *Celtis sinensis* Pers

### Sample analysis and methods

pH was determined by the potentiometric method (water:soil = 2.5:1); total phosphorus (TP) and total potassium (TK) were determined by NaOH dissolution; alkali-hydrolyzable nitrogen (AN) was determined by the alkali diffusion method; available phosphorus (AP) was determined by the 0.5 mol L^−1^ NaHCO_3_ method; available potassium (AK) was determined by ammonium acetate leaching flame photometry; soil moisture content (SMC) was determined by dehydration; soil bulk—density (BD) and total pore porosity (STP) were determined by the ring knife method; and soil mechanical composition was determined by the hydrometer method^24,25^. The classification of soil particles was according to the international system^26^ [sand (Sa): 2–0.02 mm, silt (Si): 0.02–0.002 mm, and clay (Cl): < 0.002 mm]. A more detailed explanation of the methodology is provided in Sparks et al.^27^.

The detection of soil organic nitrogen (SON) components will be carried out using the Bremner^4^ method, please refer to Table 2.

**Table 2:** Detection methods of soil organic nitrogen components.

Nitrogen component	Operation Steps
Preparation of acid hydrolysis solution	1. Weigh 2.0000 g of the soil sample into a 150 mL Erlenmeyer flask, and add 2 drops of n-octanol and 20 mL of 6 mol/L HCl, then shake it 2. Let it stand on an electric hot plate at 120 °C for 12 h until it refluxes, and screen it after cooling 3. Pour the filtrate into a 60 mL beaker, wash the residue with water until the volume is nearly 60 mL. Add NaOH to adjust the pH to 6.5 ± 0.3 (operate below 20 °C and add NaOH when it is not alkaline). Make up the volume to 100 mL and store it at 4 °C
Total acid-hydrolyzable nitrogen (TAN)	1. Take 5 mL of the acid hydrolysis solution into a Kjeldahl tube, and add 3 mL of H₂SO₄ and a catalytic reagent for digestion 2. Heat it on an electric furnace until there are no bubbles and it turns green, then heat it for another 1 h. Add 10 mL of 10 mol/L NaOH for distillation and determination
Acid-hydrolyzable ammonia nitrogen (AMN)	Take 5 mL of the acid hydrolysis solution into a Kjeldahl tube, and add 0.07 ± 0.01 g of MgO for distillation measurement
Acid-hydrolyzable amino acid nitrogen (AAN)	1. Take 5 mL of the acid hydrolysis solution into a Kjeldahl tube, add 1 mL of 0.5 mol/L NaOH, and heat it in boiling water for 20 min, then cool it down 2. Add 0.1 g of ninhydrin hydrate and 0.5 g of citric acid. Heat it in a water bath for 1 min, shake it gently for 9 min, and then let it cool 3. Add 10 mL of buffer solution and 1 mL of 5 mol/L NaOH for distillation detection
Acid-hydrolyzable ammonia nitrogen (AMN) and amino sugar nitrogen (ASN)	Take 10 mL of the acid hydrolysis solution into a Kjeldahl tube, add 10 mL of phosphate solution to adjust the pH to 11.2, and then conduct distillation determination
Other types of nitrogen	1. Non-Acid-Hydrolyzable Nitrogen (AIN) = Total Nitrogen (TN)—Total Acid-Hydrolyzable Nitrogen (TAN); 2. Amino Sugar Nitrogen (ASN) = (Ammonia Nitrogen (AMN) + Amino Sugar Nitrogen (ASN))—Ammonia Nitrogen (AMN); 3. Unknown Nitrogen (TUN) = Total Acid-Hydrolyzable Nitrogen (TAN)—(Ammonia Nitrogen (AMN) + Amino Sugar Nitrogen (ASN) + Acid-Hydrolyzable Amino Acid Nitrogen (AAN)), calculated by the difference method

### Statistical methods

One-way analysis of variance (ANOVA) was used to systematically investigate the effects of vegetation restoration on soil organic nitrogen (ON) fractions and physicochemical properties. Redundancy analysis (RDA) was employed to explore the relationships between organic nitrogen fractions and environmental factors. RDA can simultaneously analyze the responses of multiple organic nitrogen fraction variables to environmental factors, maximizing the covariance to reveal potential patterns. Subsequently, a Monte Carlo permutation test was conducted to rank the importance of ecological factors. This non—parametric test generates a null distribution by randomly permuting the data matrix multiple times. By comparing the test statistics of the observed and permuted data, the significance and importance of each factor are determined, and the key environmental factors are identified. A Mantel test was carried out to evaluate the significance of the influence of various factors on organic nitrogen fractions. This test calculates the correlation between the distance matrices of samples to determine the relationship between the data patterns. In terms of analysis implementation, ANOVA was performed in SPSS 26.0; RDA and the Monte Carlo permutation test were executed in Canoco 5.0, which specializes in community ecological analysis. The Mantel test and visualization were achieved with the dplyr, ggcor, and ggplot2 packages in R v4.2.2, which are used for data processing, calculating the correlation matrix, and creating visual charts, respectively.

## Results and analysis

### Changes in organic nitrogen fractions

The contents of organic nitrogen fractions at different restoration durations all decreased with the increase of soil depth. The content in the 0–20 cm soil layer was higher than that in the 20–40 cm soil layer, showing an obvious surface accumulation phenomenon.

**Fig. 2:**
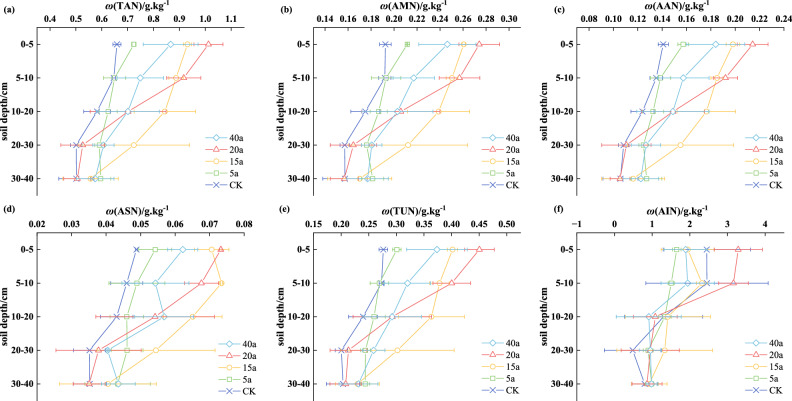
Effect of vegetation restoration on soil nitrogen fractions. (CK: farmland, 5 a: grassland, 15 a: shrub-grassland, 20 a: shrubland, 40 a: forestland, same below).

Except for non-acid-hydrolyzable nitrogen (AIN), as the vegetation restoration duration increased, in the 0–10 cm soil layer, the order of organic nitrogen content was as follows: 20-year-old shrubland > 15-year-old shrub-grassland > 40-year-old forestland > 5-year-old grassland > control (CK). In the 10–40 cm soil layer, the order was: 15-year-old shrub-grassland > 20-year-old shrubland > 40-year-old forestland > 5- year-old grassland > control (CK). Notably, in the 0–40 cm soil layer, the 15-year-old shrub-grassland had the highest organic nitrogen content. The proportions of its total acid-hydrolyzed nitrogen (TAN), acid-hydrolyzed ammonium nitrogen (AMN), acid—hydrolyzed amino acid nitrogen (AAN), acid-hydrolyzed amino sugar nitrogen (ASN), and acid-unknown nitrogen (TUN) were 26.70%, 22.80%, 26.08%, 31.50%, and 28.77% higher than those of the control (CK), respectively. In the 0–40 cm soil layer, the contents of non-acid-hydrolyzable nitrogen (AIN) in the 20-year-old shrubland and 15-year-old shrub-grassland were 19.8% and 5.66% higher than those in the cultivated land, respectively. The maximum values of TAN, AMN, AAN, ASN, TUN, and AIN all occurred in the 0–5 cm soil layer. With the increase of depth, the maximum decreases were 57.95%, 50.44%, 60.99%, 67.06%, 63.04%, and 98.86%, respectively. The results indicated that vegetation restoration increased the soil organic nitrogen fractions (Fig. 2).

### Soil organic nitrogen fraction as a proportion of total nitrogen

**Fig. 3:**
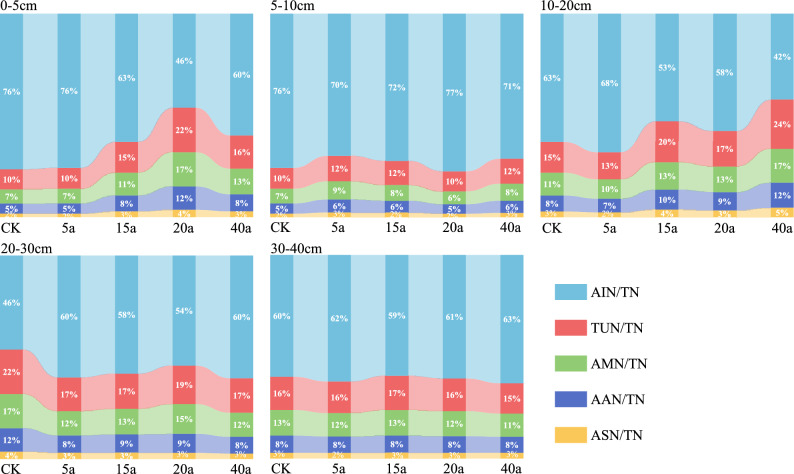
Soil organic nitrogen as a percentage of total nitrogen.

As the vegetation restoration duration increases, the changes in soil organic nitrogen (ON) content can be used to characterize the relative proportions of various organic nitrogen fractions (Fig. 3). Non-acid-hydrolyzable nitrogen (AIN) and acid-hydrolyzed amino sugar nitrogen (ASN) account for the highest and lowest proportions of organic nitrogen, respectively. The relative proportions of different organic nitrogen fractions to total nitrogen (TN) ranked as follows: AIN (46%-76%; mean 64.64%) > TUN (10%-24%; mean 15.6%) > AMN (7%-17%; mean11.48%) > AAN (5%-12%; mean 7.92%) > ASN (2%-5%; mean 2.92%). The proportion of soil non-acid-hydrolyzable nitrogen decreases with the increase of soil depth, while the proportions of other forms of nitrogen increase. Among them, non-acid-hydrolyzable nitrogen (AIN) is the main component of organic nitrogen.

In the 0–5 cm soil layer, different restored vegetation types ranked by acid—hydrolyzable nitrogen content are as follows: 20-year-old shrubland > 40-year-old forestland > 15-year-old shrub-grassland > 5-year-old cultivated land. In this soil layer, the acid-hydrolyzable nitrogen content of the 20-year-old shrubland is 19.74% higher than that of the control (CK), while its non-acid-hydrolyzable nitrogen content is lower.

In the 5–20 cm soil layer, the acid-hydrolyzable nitrogen content shows an increasing trend, while the non-acid-hydrolyzable nitrogen content shows a decreasing trend. However, in the 10–20 cm soil layer, the situation changes: the non- acid-hydrolyzable nitrogen content increases, while the contents of other forms of nitrogen decrease. In this 10–20 cm layer, different restored vegetation types ranked by acid-hydrolyzable nitrogen content are: 40-year-old forestland > 15-year-old shrub -grassland > 20-year-old shrubland > farmland > 5-year-old grassland. In the 20–30 cm soil layer, the content of soil non-acid-hydrolyzable nitrogen (AIN) increases, while the acid-hydrolyzable nitrogen content decreases. In the 30–40 cm soil layer, the acid-hydrolyzable nitrogen content tends to be stable. Compared with the control, vegetation restoration increases the proportion of acid-hydrolyzable nitrogen and decreases the proportion of non-acid-hydrolyzable nitrogen, respectively.

### Effects of environmental factors on soil organic nitrogen fractions

Soil physical and chemical properties have a significant impact on soil nitrogen fractions. Therefore, in this study, statistical analysis was conducted. Specifically, total acid-hydrolyzed nitrogen (TAN), acid-hydrolyzed amino acid nitrogen (AAN), acid-hydrolyzed ammonium nitrogen (AMN), acid-hydrolyzed amino sugar nitrogen (ASN), acid-unknown nitrogen (TUN), and non-acid-hydrolyzable nitrogen (AIN) were used as dependent variables, while soil organic carbon (SOC), pH, bulk density (BD), soil total phosphorus (STP), chlorine (Cl), silicon (Si), sand content (Sa), soil moisture content (SMC), total phosphorus (TP), total potassium (TK), total nitrogen (TN), alkali-hydrolyzable nitrogen (AN), available phosphorus (AP), available potassium (AK), ammonium nitrogen (NH₄⁺-N), and nitrate nitrogen (NO₃⁻-N) were used as independent variables (explanatory variables).

**Fig. 4:**
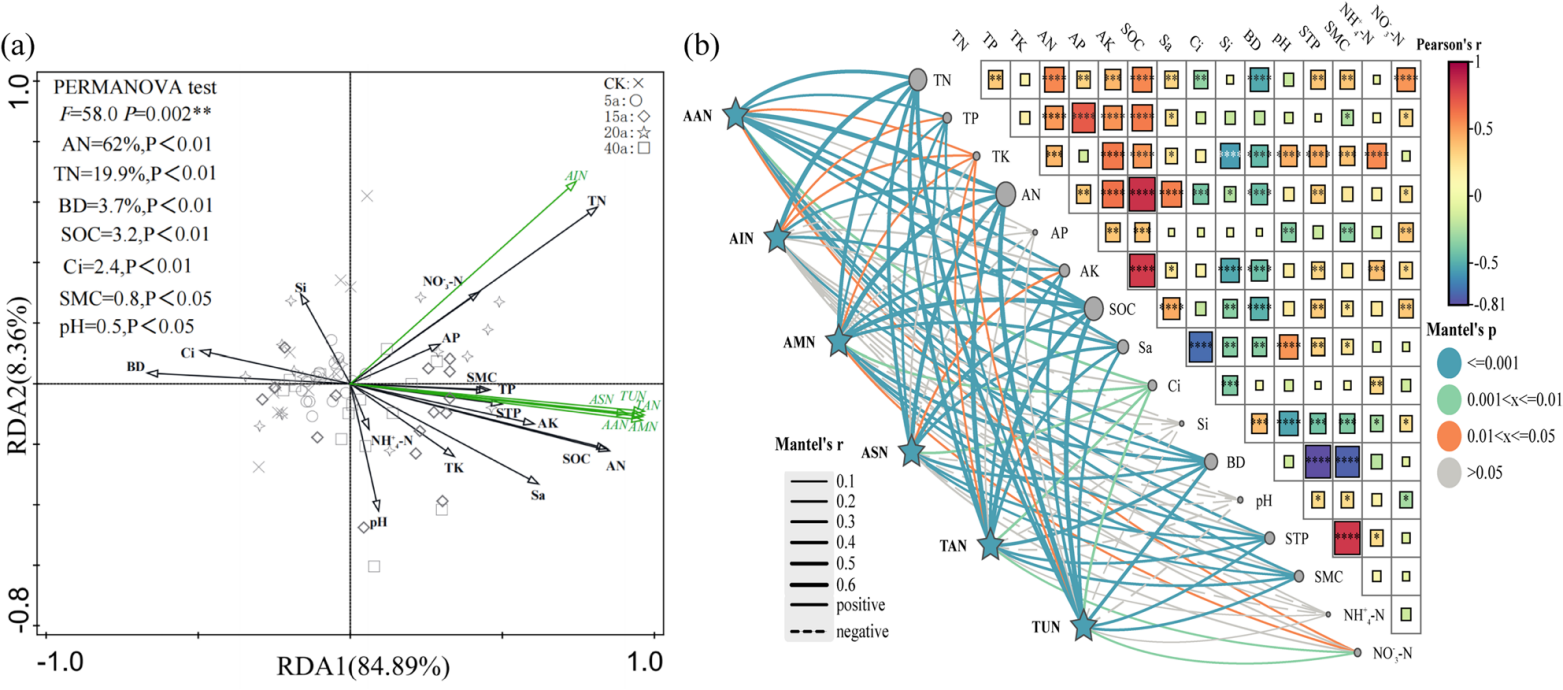
Effect of environmental factors on soil organic nitrogen fractions. (TAN acid-hydrolyzed total nitrogen, AAN amino acid nitrogen, AMN acid-ammoniacal nitrogen, ASN aminoglycan nitrogen, TUN acid-unknown nitrogen, AIN non-acid-hydrolyzed nitrogen; SOC organic carbon, pH, BD bulkiness, STP total porosity, Cl viscous grains, Si pulverised grains, Sa sandy grains, SMC water content, TN total nitrogen, AN alkali-dissolved nitrogen, AP quick-acting phosphorus, AK quick-acting potassium, NH_4_^+^-N ammoniacal nitrogen and NO_3_^-^-N nitrate nitrogen, TN). (Rectangles are correlation heatmaps of soil physicochemical factors, the thickness of the line represents the size of the correlation coefficient of the Mantel,s r test (solid line positive correlation, dashed line negative correlation), and the colour of the line indicates that the value of the Mantel,s p test (the line denotes the statistical significance) pairs organic nitrogen fractions with the environmental factors (orange *P < 0.05, cyan **P < 0.01, blue ***P < 0.001).

Figure 4a shows the results of redundancy analysis (RDA) and the Monte Carlo test. As shown in Fig. 4a, the first two axes cumulatively explained 93.25% of the variation in soil organic nitrogen fractions. AN, TN, and SOC were extremely significantly correlated with organic nitrogen fractions, cumulatively explaining 85.1% of the observed variation. In contrast, Cl, Si, and BD were negatively correlated with these fractions. Specifically, AIN was negatively correlated with pH, NH₄⁺-N, Cl, Si, and BD.

As shown in Fig. 4b, the Mantel test results indicated that acid—hydrolyzed nitrogen (including AMN, AAN, ASN, TUN) and TAN were extremely significantly correlated with TN, TP, AN, SOC, Sa, BD, STP, and SMC, but showed no correlation with Si, pH, AP, and NH₄⁺-N. Except for ASN, which had no correlation with TK and Cl, acid-hydrolyzed nitrogen was correlated with all the other factors. AIN, on the other hand, was extremely significantly correlated with TN, AN, SOC, BD, TP, and TK, yet it was not correlated with Si, pH, AP, and NH₄⁺-N.

Overall, in the study area, AN, TN, and SOC had the most significant impact on soil organic nitrogen fractions, while Si and pH showed no correlation with these fractions.

## Discussion

The mechanism by which vegetation changes influence the component content of soil organic nitrogen, whose structure and components are complex, is often attributed to environmental factors^28^, soil properties^29^, and plant growth status^12^. Our study found that under different restoration durations, the content of each soil organic nitrogen component decreased with the increase of soil depth, showing an obvious surface-accumulation phenomenon. Except for non-acid-hydrolyzable nitrogen (AIN), the contents of other organic nitrogen components were higher than those in the control group (CK) (Fig. 2). This indicates that vegetation cover and restoration are beneficial to the accumulation of soil nitrogen nutrients^30,31^. The reason is that during vegetation restoration, the decomposition of litter and the release of root exudates provide additional nitrogen sources for the soil, increasing soil nitrogen input^32,33^. Acid-hydrolyzed ammonium nitrogen (AMN) and acid -hydrolyzed amino acid nitrogen (AAN) are the main forms of soil organic nitrogen. Their contents are linearly correlated with the mineralization rate of organic nitrogen and are regarded as the main sources of easily mineralizable organic nitrogen^34–36^. Our study shows that the content of acid-hydrolyzed ammonium nitrogen (AMN) in the 15-year-old shrubland is generally higher than that in other restoration durations. This indicates that its soil nitrogen supply capacity is relatively strong.

In addition, acid—hydrolyzed amino acid nitrogen (AAN) is the main source of available nitrogen for soil microorganisms and current-season plants and is closely related to microbial metabolism^10,37^. It is often used to characterize the soil nitrogen supply potential^38^. In this experiment, the ratio of the acid-hydrolyzed amino acid nitrogen (AAN) content to the total nitrogen content in soils of different restoration durations was 5–12%, and there was no significant difference among soil layers. This indicates that there was no significant difference in the soil nitrogen supply potential among different restoration durations.

Moreover, the mass fraction of acid-hydrolyzed amino sugar nitrogen (ASN) showed no obvious difference under different restoration durations, which is consistent with previous studies^9,34^. The study by Wang et al.^39^ showed that acid—hydrolyzed amino sugar nitrogen (ASN) was not correlated with soil total nitrogen and mineral nitrogen. The ratios of AIN/TN and acid—unknown nitrogen (TUN)/TN in the soil were relatively high, which is consistent with the research results of He et al.^40^. The composition of acid—unknown nitrogen (TUN) is complex. It is generally considered to be related to humic acid, fulvic acid, and microbial products^41^, mainly containing heterocyclic organic nitrogen and humus^11^. It accumulates easily in the soil due to its slow mineralization^42^.

The chemical form of soil non-acid-hydrolyzable nitrogen is stable. It can produce stable humus, is not easily mineralized, and is not conducive to improving the comprehensive soil nitrogen supply level^43^. Studies have shown that compared with cultivated land, vegetation restoration helps to reduce the proportion of soil non-acid-hydrolyzable nitrogen in total nitrogen (Fig. 3). In the soil of the 20-year-old shrubland, the content of non-acid-hydrolyzable nitrogen was significantly higher than that in soils of other restoration durations and the control group. As a persistent form of nitrogen, the increase in the content of non-acid-hydrolyzable nitrogen helps to improve the soil nitrogen retention capacity and reduce nitrogen loss in natural ecosystems^44^. Although an increase in the proportion of non-acid-hydrolyzable nitrogen will reduce the mineralization rate of soil organic nitrogen, it is beneficial to improving the soil nitrogen storage capacity^5^.

Most soil organic nitrogen exists in the form of polymers^11^. Bacteria can decompose it into small molecules such as amino acids and sugars. Under suitable temperature, humidity, and organic matter conditions, the decomposition effect of bacteria on organic nitrogen (ON) is enhanced. When decomposing ON, microorganisms use it as a carbon source^45^, but this process is regulated by soil temperature, moisture, pH, and soil organic matter. Both biological and abiotic factors can regulate soil nitrogen components. The present study shows that among the investigated factors significantly positively correlated with acid-hydrolyzed nitrogen components, total nitrogen (TN), alkaline-hydrolyzable nitrogen (AN), and soil organic carbon (SOC) had the highest correlations (Fig. 4), which is consistent with the research results of Pan et al.^46^. The quality and quantity of soil total nitrogen directly affect the ease with which microorganisms involved in nitrogen mineralization obtain the energy and nitrogen sources required for ON decomposition, thereby affecting soil nitrogen transformation characteristics^47^. Soil organic carbon provides sufficient energy for soil microorganisms and promotes the mineralization of soil nitrogen by microorganisms^48^. Among the investigated factors, the contents of soil organic carbon, total nitrogen, and alkaline-hydrolyzable nitrogen had the greatest promoting effect on nitrogen mineralization, which is consistent with previous research results^49^.

In this study, the contents of AAN, AMN, ASN, TUN were extremely significantly positively correlated with the content of TAN (Fig. 4), possibly because they are all important components of TAN. At the same time, compared with AAN and ASN, TUN and AMN contributed more to TAN, indicating that TUN and AMN may be the main sources of available nitrogen for plant uptake. This experiment also shows that soil organic carbon was extremely significantly correlated with organic nitrogen. Vegetation restoration increases exogenous organic carbon, provides sufficient energy for nitrogen transformation, directly affects the biomass and activity of soil microorganisms^12^, and regulates the content and availability of various forms of nitrogen in the soil. The combined effect of environmental factors and soil nutrient input affects the composition and distribution of soil acid-hydrolyzed nitrogen. The changes in each component of soil organic nitrogen may be due to the fact that vegetation restoration increases the content of soil organic carbon and enhances microbial activity, thereby causing changes in each component of organic nitrogen.

## Conclusions

Under different restoration durations, the contents of each soil organic nitrogen component (except for AIN) are higher than those in the control group, showing a surface-accumulation phenomenon. Vegetation restoration increases nitrogen input through the decomposition of litter and the release of root exudates, which is beneficial to the accumulation of soil nitrogen nutrients. In terms of the characteristics of organic nitrogen components, AMN and AAN are the main sources of easily mineralizable organic nitrogen. The AMN content in the 15-year-old shrubland is high, indicating strong soil nitrogen supply capacity. Moreover, the nitrogen supply potential of soil AAN shows no significant difference among different restoration durations. In addition, the mass fraction of ASN shows no obvious difference under different restoration durations and has no correlation with total soil nitrogen and mineral nitrogen. The TUN has a complex composition, a slow mineralization rate, and is easy to accumulate. The ratios of AIN to TN and TUN to TN are relatively high.

Both biological and abiotic factors regulate soil nitrogen components. TN, AN, SOC are significantly and positively correlated with acid-hydrolyzed nitrogen components and have a great promoting effect on nitrogen mineralization. The contents of AAN, AMN, ASN, and TUN are extremely significantly and positively correlated with the content of TAN. Thus, TUN and AMN may be the main sources of available nitrogen for plant uptake. Finally, vegetation restoration increases organic carbon, affects microbial activity, and thus regulates the content and availability of soil nitrogen.

## Supplementary Information

Supplementary Information.

## Data Availability

All data generated or analysed during this study are included in this published article [and its supplementary information files].
